# Intermediate outcomes in randomized clinical trials: an introduction

**DOI:** 10.1186/1745-6215-14-78

**Published:** 2013-03-19

**Authors:** Armando H Seuc, Alexander Peregoudov, Ana Pilar Betran, Ahmet Metin Gulmezoglu

**Affiliations:** 1 Reproductive Health Research Department, World Health Organization, Geneva 27 1211, Switzerland

**Keywords:** Intermediate outcomes, Intention-to-treat approach, Principal stratification, Causal effects

## Abstract

**Background:**

Intermediate outcomes are common and typically on the causal pathway to the final outcome. Some examples include noncompliance, missing data, and truncation by death like pregnancy (e.g. when the trial intervention is given to non-pregnant women and the final outcome is preeclampsia, defined only on pregnant women). The intention-to-treat approach does not account properly for them, and more appropriate alternative approaches like principal stratification are not yet widely known. The purposes of this study are to inform researchers that the intention-to-treat approach unfortunately does not fit all problems we face in experimental research, to introduce the principal stratification approach for dealing with intermediate outcomes, and to illustrate its application to a trial of long term calcium supplementation in women at high risk of preeclampsia.

**Methods:**

Principal stratification and related concepts are introduced. Two ways for estimating causal effects are discussed and their application is illustrated using the calcium trial, where noncompliance and pregnancy are considered as intermediate outcomes, and preeclampsia is the main final outcome.

**Results:**

The limitations of traditional approaches and methods for dealing with intermediate outcomes are demonstrated. The steps, assumptions and required calculations involved in the application of the principal stratification approach are discussed in detail in the case of our calcium trial.

**Conclusions:**

The intention-to-treat approach is a very sound one but unfortunately it does not fit all problems we find in randomized clinical trials; this is particularly the case for intermediate outcomes, where alternative approaches like principal stratification should be considered.

## Background

The presence of intermediate outcomes (IO), both in experimental and observational research, complicates the assessment of causal effects between exposures and (final) outcomes [1]. Methodological and statistical approaches for dealing with this problem have been proposed for quite a time [2-5], but many researchers are not yet familiar with them. The purpose of this article is to present an intuitive introduction to a framework for studying causal effects when IOs are present, principal stratification (PS), and a related statistical technique, instrumental variables (IV). We illustrate concepts and methods using the World Health Organization (WHO) randomized trial of calcium supplementation before pregnancy to reduce recurrent preeclampsia [6].

Before describing the techniques we present a summary of the calcium trial, and a few basic definitions that are required to understand them. We expect that this paper will help researchers conducting randomized clinical trials (RCT) to become more familiar with these techniques, and that their proper application will be further promoted.

## Methods

### Summary description of the calcium supplementation trial

Calcium supplementation has been shown to reduce severity of preeclampsia, maternal morbidity and newborn mortality when supplementation starts at around mid-pregnancy, and particularly in women with low calcium intake [6]. However, calcium supplementation in the second half of pregnancy may be too late to affect pre-eclamptic processes, and it has been proposed that further improvements in outcomes may be achieved by earlier supplementation. Consequently, the WHO randomized trial of calcium supplementation before pregnancy to reduce recurrent preeclampsia [6] aims at assessing if calcium supplementation before and in the first half of pregnancy reduces the incidence of recurrent preeclampsia more effectively than supplementation starting at 20 weeks.

The trial will be conducted in maternities in South Africa, Zimbabwe and Argentina. Participants in the trial will be women who have had preeclampsia or eclampsia in their most recent pregnancy and are planning to become pregnant. The trial will randomize 1,410 subjects, and it is expected that about 700 women (half) will get pregnant, 350 in each trial group. The estimated duration of the trial is four years over which 540 pregnant women (270 in each trial group) are estimated to remain in the sample and will be included in the final analysis. The primary outcome of the trial will be incidence of preeclampsia.

The study group will receive calcium supplementation with 500 mg elemental calcium daily from enrolment (before pregnancy) until 20 weeks’ gestation, and the control group will receive placebo. All women will receive calcium supplementation (1.5 mg) unblinded from 20 weeks’ gestation until delivery. If pre-pregnancy calcium supplementation is found to be effective, the groundwork will have been done for research and then implementation of food fortification programs. The possibility for calcium supplementation to affect the risk of pregnancy is very low, but it cannot be ruled out. The protocol has been approved by the institutional review boards of the participating centres. All of the participants will provide written informed consent.

### Some relevant definitions

**Figure 1:**
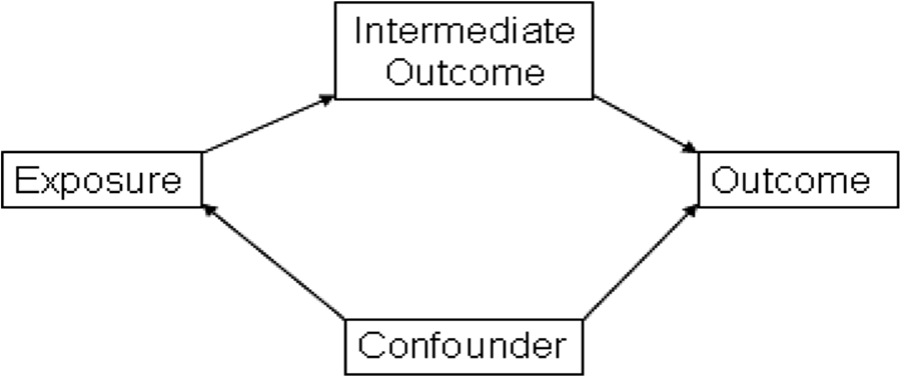
Difference between confounders and intermediate outcomes.

In epidemiology, an IO (sometimes known as a mediating variable) is ‘any factor that represents a step in the causal chain between the exposure and disease [that] should not be treated as an extraneous confounding factor, but instead requires special treatment’ [7,8]. In causal terms, the confounder is expected to cause the exposure, but the exposure will cause the intermediate outcome [7]. See Figure 1.

For example, in the WHO calcium supplementation trial described above, compliance to treatment assigned (calcium or placebo) is an intermediate outcome, not a confounder, between assignment to calcium and preeclampsia (or even pregnancy). As subjects in the calcium trial will be randomly assigned to calcium we will in general not have confounders as nothing can cause randomization; this systematic tendency to avoid bias (from confounding) is one of the main reasons why results from RCTs are more credible than those from observational studies.

Also in this context, pregnancy is a (potential) intermediate outcome, not a confounder, between treatment assigned (or treatment received) and preeclampsia. If there were theoretical and/or empirical evidence that indeed calcium influences the risk of pregnancy, then pregnancy would be an intermediate outcome; if the risk of pregnancy were independent of calcium intake, then pregnancy would not be an intermediate outcome. In this last scenario, the subgroup of pregnant women in each of the two trial arms would be a random sample, no selection bias would be present and the effect of calcium supplementation on preeclampsia could safely be conducted comparing the corresponding subgroups of pregnant women.

If the intermediate outcome also causes exposure, then it is also a confounder*.* This is essentially possible only in observational studies, as in experimental studies it is usually possible to identify the direction of the causal effect. For example, in an observational cohort study considering the effect of exposure to tobacco and alcohol on heart diseases, the two-way dependency between tobacco and alcohol makes each of them a confounder and also an intermediate outcome, with respect to the outcome heart disease.

Other relevant related terms are endogenous variable and instrumental variable, traditionally used in the context of non-experimental studies. An endogenous explanatory variable is an explanatory variable in a regression model that is correlated with the error term, either because of an omitted variable, measurement error, or the explanatory variable is (also) determined by the dependent variable (reverse causality) [7].

An IV is a variable not included in the regression model, which is uncorrelated with the error term (that is, uncorrelated with the dependent variable) and is (partially) correlated with the endogenous explanatory variable [7,9]. IVs have a long history within econometrics but are not yet widely used in the health sciences [10,11].

To illustrate the term endogenous variable let us suppose that in our calcium trial example we try to predict pregnancy using treatment received (calcium/placebo) as a predictor; then treatment received is in general an endogenous explanatory variable because of the potential selection bias that noncompliance (from being randomized to effectively taking calcium) might introduce. Those who do receive calcium are in general not comparable to those who do not, which means that we need other predictors to properly model the response variable pregnancy, making treatment received an endogenous variable.

To illustrate the term IV in the context of RCTs, let us follow up the previous example where treatment received was an endogenous variable; in this scenario treatment assigned is an IV because i) it does (potentially) effect pregnancy only through effective calcium intake, and ii) calcium intake is (positively) correlated to calcium assignment. Then this treatment assigned IV is used to remove or mitigate the endogeneity of treatment received as a predictor for pregnancy; this is done using, for example, two-stage least squares (TSLS) regression.

Random assignment to treatment is frequently used in RCTs as an IV when we are interested in the causal effect of treatment (effectively) received on the (final) outcome, as it helps to obtain less biased causal effect estimations in the presence of significant lack of treatment compliance [9]. IV analyses are frequently conducted in observational studies; in these cases it relies on finding an IV, which is usually a naturally varying phenomenon, related to treatment but not to outcome, except through the effect of treatment itself, and then using this phenomenon as a proxy for the confounded treatment variable. In doing so the IV analysis of observational studies parallels the RCT design [12].

An intuitive justification for finding and using IV is that under certain conditions the association of interest between exposure (treatment received) *R* and outcome *Y*, Assoc _RY_, can be written as:

(1) $${\text{Assoc}}_{\mathrm{TY}}={\text{Assoc}}_{\mathrm{TR}}*{\text{Assoc}}_{\mathrm{RY}}.$$

where *T* is the IV; solving for the association between *R* and *Y* in (1) provides a better estimate of the relevant parameter than the unadjusted (or crude) association, when conditions i) and ii) above are met [11]. In the above examples we can observe that IVs become the new (proxy) exposures, while the original exposures are recycled as IO.

Finally, trials can be classified as pragmatic trials or explanatory trials. Pragmatic trials are those designed to assess the effectiveness of a treatment in routine, everyday practice; on the other side explanatory trials are those designed to assess the efficacy of a treatment (usually compared with placebo) under ideal, experimental conditions [13]. Pragmatic trials and the ITT approach are naturally connected as the last one focuses on the treatment allocated and not on what and/or how the treatment was really taken, which is precisely what typically occurs in routine, everyday practice. However, the standard application of the ITT approach is possible only when complete outcome data are available for all randomized subjects, and it requires explicit description of the handling of deviations from randomized allocation and missing responses [14].

### Problems caused by intermediate outcomes

The intention-to-treat (ITT) effect is not a causal effect; it is the effect of being randomized to the experimental (or active) treatment in comparison to the control treatment [15]. On some occasions IOs make this ITT effect irrelevant, or at least secondary. For example, due to noncompliance the effect of being assigned the experimental treatment and actually receiving it can be very different, and we might be interested mostly (or also) in the last one (the causal effect). In other cases, the final outcome is not defined for certain values/categories of the IO, and the ITT effect is useless; for example in our calcium trial the final outcome, preeclampsia, is not defined for women who do not get pregnant, and the ITT would force the analysis to consider preeclampsia in non-pregnant women as zero (or some other unjustified and surely inappropriate value). It should be noted also that using regression models to adjust for IOs is not the solution, mainly because this approach forces us to base the decision to treat (with the experimental treatment) on information not available at the required time (which is emulated within a RCT by the time when randomization is made).

Within the context of an RCT, the ITT is (almost) always unbiased, while the effect derived from the as-treated analysis (where subjects are grouped and compared according to the treatment actually received) is generally biased; the ITT effect estimates the effect of being randomized to the experimental treatment, while the as-treated effect estimates the effect of actually receiving the experimental treatment. So if we are interested in the causal effect it could be argued that, ultimately, it might be better to have a biased answer to the right question than a perfect answer to the wrong question. Fortunately, due to appropriate statistical techniques, we can have a close-to-unbiased answer to the question about the relevant causal effect.

As in many other research areas, noncompliance and missing data are two rather common IOs in reproductive health research RCTs. For example in a recent Cochrane Review of calcium supplementation during pregnancy for preventing hypertensive disorders and related problems [16], four out of thirteen trials included in the review had significant noncompliance (15% or more), and five out of thirteen had 5% or more lost to follow up. In the case of noncompliance, three trials [17-19] approached this problem using (modified versions of) ITT, and in one trial [20] it was approached using sensitivity analysis.

It is also frequent to have IOs generating so-called truncation by death (or partially defined final outcomes [21]) as in the case of the calcium trial, where the final outcome preeclampsia is not defined if the woman does not get pregnant. It should be noted that noncompliance is an IO that does not prevent us from observing the final outcome, while missing data does (although in this last case the missing outcomes are potentially observable and can eventually be imputed); this illustrates very clearly that there are relevant differences between various IOs.

### Principal stratification and instrumental variables for intermediate outcomes

PS is a conceptual framework developed in the setting of counterfactual causal inference to deal with situations where the causal path from the treatment to the outcome includes an IO variable that cannot be ignored [5]. PS can be viewed as having its seeds in the IV method, which originated in non-experimental econometric studies a long time ago; under the PS approach the IV method has been re-interpreted as a way to approach a randomized experiment that suffers from IOs [22].

Our presentation in these sections borrows, among others, from Stuart *et al*. [15] and Yau and Little [23]. Stuart *et al*. [15] make a comprehensive non-technical presentation of PS and IV in the context of noncompliance as an IO, and the paper by Yau and Little [23] describes how to account for more than one IO in the same trial, specifically noncompliance and missing data. Let us consider a general situation where each member i of a set of N subjects has been randomly assigned to either an experimental treatment (*Ti* = 1) or a control treatment (*Ti* = 0). If there are k ordered intermediate outcomes *IO*_*1*_*, IO*_*2*_*, …, IO*_*k*_, and all of them are binary (*IO*_*j*_ *=* 1 or 0 for all *j*), we will denote by:

$$\mathrm{Pi}=\left(IO_{1i},IO_{2i},\ldots ,IO_{\mathrm{ki}}\right)$$

the k-dimension vector with the observed profile for subject *i*, out of 2^k^ possible profiles. If the k intermediate outcomes are not ordered then there is a possible maximum total of 2^k^ × k! profiles. For example in our calcium trial the IOs, pregnancy, and missing, are in general not ordered in the sense that they can occur in different sequences (missin,g either before pregnancy or after pregnancy); accounting for this complication means that there would be a total of 2^2^ × 2! = 8 profiles.

Finally let us assume that each subject has two potential (final) outcomes: *Yi* (*Ti = 1, Pi*), the outcome we would observe if subject *i* is assigned to the treatment group and has profile *Pi*, and *Yi* (*Ti* = 0, *Pi*), the outcome we would observe if subject *i*, is assigned to the control group and has profile *Pi*; it should be noted however that for subjects in some profiles the *Yi* outcome might not be (sufficiently) defined due to the interference of IOs generating truncation by death or partially defined final outcomes [21].

The ITT effect of the treatment for subject, *i*, is defined as the difference in subject *i*’s outcomes if assigned to experimental versus control treatment:

$$\tau_{i}=\mathrm{Yi}\left(\mathrm{Ti}=1,\mathrm{Pi}\right)–\mathrm{Yi}\left(\mathrm{Ti}=0,\mathrm{Pi}\right)$$

For each subject, however, we are able to observe only one of these two potential outcomes. Some authors consider potential outcomes and counterfactuals as exchangeable, but as pointed about by Rubin [24], it is safer to keep a distinction in the sense that after assignment (and a particular profile of IOs) one of the potential outcomes is observed while the other turns a counterfactual, while before assignment they were not counterfactuals.

For subjects in the experimental group we observe *Yi* (*Ti* =1, *Pi*) whereas for subjects in the control group we observe *Yi* (*Ti* = 0, *Pi*). We can, however, estimate the overall average treatment effect (the ITT effect), which is a comparison of outcomes if everyone was assigned to the treatment group versus everyone assigned to the control group:

(2) $$\tau =\frac{1}{N}\overset{N}{\underset{i=1}{\sum }}Y_{i}\left(T_{i}=1,P_{i}\right)-Y_{i}\left(T_{i}=0,P_{i}\right)$$

Given our previous assumption of binary treatment assignment, and the k binary and ordered IOs, there are then 2 × 2^k^ = 2^k+1^ profiles that can be properly combined to form (principal) strata; each stratum is created to group together subjects with a particular profile *Pi* both under treatment and under control (*Ti* =1 and *Ti* = 0, respectively), so in general there will be also 2^k+1^ principal strata. For example in the calcium trial the possible (ordered) IOs compliance, pregnancy, and missing, would generate 2^3+1^ = 16 principal strata as a result of considering the eight profiles from IOs under the two possible assignments (to treatment and control).

Therefore, we can also express the overall ITT effect as the (weighted) average of the ITT effects for each of these 2^k+1^ strata, that is:

(3) $$\tau =p_{1}\tau_{1}+p_{2}\tau_{2}+\ldots +p_{2^{k+1}}\tau_{2^{k+1}},$$

with *p*_*j*_ the proportion of the population in stratum j, and τ_j_ the corresponding average treatment assignment effect.

Two important properties of this stratification using potential outcomes, known as PS, are: i) it stratifies on the indicators of the IOs (observed at randomization and not affected by it), but not on the observed IOs, which are outcomes generally affected by the treatment received [25], and ii) we might find that at least for one of these strata, the effect of assignment to the experimental treatment (the ITT effect) is (almost) the same as the relevant causal effect.

For example, in the calcium trial and considering the three ordered IOs (compliance, pregnancy, and missing), property ii) above is illustrated by considering the specific stratum non-missing, always pregnant, and compliers; the ITT effect for this principal stratum is (close to) what we could call the causal effect of calcium on preeclampsia.

In the next sections we will describe PS and IV in the context of two frequent IOs in reproductive health research, namely, noncompliance and pregnancy. Section “Compliance as intermediate outcome” presents the PS approach, and the Sections “Assumptions underlying CACE analyses”, “Simple description of estimation” and “More complex estimation” describe the simple version of the IV technique within the specific context of noncompliance as the only IO. We also discuss the specific characteristics of the truncation by death (or partially defined) type of IO, and why the simple IV estimate presented in Section “Compliance as intermediate outcome” might not be applicable [25].

### Compliance as intermediate outcome

As before, let us consider that each member of a set of *n* subjects has been randomly assigned to either an experimental treatment (*Ti* = 1) or a control treatment (*Ti* = 0), and let us assume some of the experimental group members actually receive the control treatment, and some of the control group members may actually receive the experimental treatment. Denote the actual treatment received (the only IO in this case) as *Pi* (that is, now having only 2^1^ = 2 profiles). As before let us consider the situation where the treatment (assigned or received) is either 0 or 1, indicating control treatment or experimental treatment. Each subject has two potential outcomes: *Yi* (*Ti* =1, *Pi*), the outcome we would observe if subject *i* is assigned to the treatment group, and *Yi* (*Ti* = 0, *Pi*), the outcome we would observe if subject *i* is assigned to the control group. As before, the ITT effect of the treatment for subject *i* is defined as:

$$\tau_{i}=\mathrm{Yi}\left(\mathrm{Ti}=1,\mathrm{Pi}\right)–\mathrm{Yi}\left(\mathrm{Ti}=0,\mathrm{Pi}\right)$$

and the overall average treatment effect (the ITT effect) as:

$$\tau =\frac{1}{N}\overset{N}{\underset{i=1}{\sum }}Y_{i}\left(T_{i}=1,P_{i}\right)-Y_{i}\left(T_{i}=0,P_{i}\right).$$

Given our assumption of binary treatment assignment and intake, there are now four types of subjects (strata), defined by their compliance behavior when assigned to the experimental treatment and their compliance behavior when assigned to the control treatment. These four strata [26] are:

1 Compliers (C), who fully take the experimental treatment when assigned to it, and fully take the control treatment when assigned to it: (*Ti, Pi*) = (1, 1) and (*Ti, Pi)* = (0, 0); for example, in a standard clinical trial of a new drug versus placebo, these are the patients who would take the drug if in the treatment group but not if they are in the control group.

2 Always-takers (AT), who fully receive the experimental treatment when in either the experimental or control group: (*Ti, Pi*) = (1, 1) and (*Ti, Pi*) = (0, 1); for example, patients who would take the drug if they are in the treatment group, and would also be able to take it if they are in the control group.

3 Never-takers (NT), who do not receive the experimental treatment when in either the experimental or control group: (*Ti, Pi*) = (1, 0) and (*Ti, Pi*) = (0, 0); for example, patients who would not take the drug if in either the treatment or control groups.

4 Defiers (D), who do not take the experimental treatment when in the experimental group, but do take the experimental treatment when in the control group: (*Ti, Pi*) = (1, 0) and (*Ti, Pi*) = (0, 1); for example, patients who would not take the drug if in the treatment group, but would take the drug if in the control group.

Therefore, we can also express the overall ITT effect as the (weighted) average of the ITT effects for each of these four types, that is:

(4) $$\tau =p_{C}\tau_{C}+p_{\mathrm{AT}}\tau_{\mathrm{AT}}+p_{\mathrm{NT}}\tau_{\mathrm{NT}}+p_{D}\tau_{D},$$

where *p*_*j*_ is the proportion of the population in stratum *j* and *τ*_*j*_ is the corresponding average treatment assignment effect.

The second of the two main properties mentioned in the previous sections means in this case that for the stratum, compliers, who do as they are told under each treatment condition, the effect of assignment to the experimental treatment (the ITT effect) is the same as the effect of (fully) receiving it (the causal effect). Thus, our interest is in the overall ITT effect as well as the effect of assignment for the compliers stratum, or compliers average causal effect (CACE).

### Assumptions underlying CACE analyses

The effect of being randomized to treatment or to control can be easily obtained comparing the average outcomes of patients in the randomized treatment and control groups. The problem in estimating the CACE is essentially a result of the inability to identify which subjects belong to each of the four principal strata defined in the previous section. For example, patients randomized to the control group who do not take the treatment can be either compliers or never-takers, and those who do take the treatment can be either always-takers or defiers; a similar unidentifiability problem occurs with those randomized to the treatment group.

If we could observe the compliance behavior of subjects when simultaneously assigned to treatment and to control we would be able to address the unidentifiability problem, but we never can observe this. The standard IV approach imposes a basic set of assumptions to help us estimate the CACE effect. Those assumptions are [15]:

1 The outcomes of each individual are not affected by the treatment assignments of any other individuals. This assumption is made in nearly all studies estimating causal effects, and is known as no interference, or the stable unit treatment value assumption (SUTVA) [27]).

2 Being given the opportunity to take the treatment was assigned randomly.

3 Being given the opportunity to take the treatment induces some individuals to actually take it. In other words, there are some compliers. This is sometimes labeled as the non-zero denominator assumption.

4 There are no defiers. This is sometimes called monotonicity, to reflect the assumption that being told (encouraged) to do something (taking active treatment or placebo) cannot make you to do the opposite. This assumption helps identify the compliers by ruling out particular behaviors.

5 There is no effect of assignment for the never-takers or for the always-takers. In other words, since being assigned to the treatment group versus the control group does not change the patient’s treatment-taking behavior, neither change their outcomes. These assumptions (one for the always-takers and one for the never-takers) are known as the exclusion restrictions.

### Simple description of estimation

Following [15], we will describe how the above assumptions can help us estimate the CACE. We are mainly interested in estimating *τ*_*C*_, the effect for compliers, as expressed in formula (3). First, the no defiers assumption (assumption 4) implies that *p*_*D*_ = 0; and second, the exclusion restrictions (assumption 5) imply that *τ*_*AT*_ = *τ*_*NT*_ = 0. This means that *ITT* = *τ* = *p*_*C*_*τ*_*C*_. So, under the above assumptions, if we can estimate *p*_*C*_ we can obtain an estimate of *τ*_*C*_, the effect of interest.

To estimate the proportion of compliers, *p*_*C*_*,* we consider the patients in the treatment group who fully participate, who could then be either compliers or always-takers. Since we have assumed that there are no defiers (assumption 4), patients in the control group who fully take the treatment (fully participate) must be always-takers; therefore we can estimate the percentage of always-takers, *p*_*AT*_, as the proportion of patients in the control group who (fully) take the treatment.

Under assumption 2 (random assignment) the proportion of always-takers should be the same in the treatment and control groups (*idem* for the compliers stratum). This means that *p*_*AT*_ can be estimated from the control group, and *p*_*C*_ + *p*_*AT*_ can be estimated from the treatment group; this allows us to estimate the proportion of compliers *p*_*C*_, and finally the CACE from *τ*_*C*_ = *ITT*/*p*_*C*_ = *τ*/*p*_*C*_.

### More complex estimation

When the previously listed assumptions are implausible, identification of the PS requires new and more complicated assumptions, and/or methods involving relevant covariates [15,25]. The IV approach in this case involves a TSLS regression, which jointly models the two processes of treatment received (IO) and the *Y* outcome observed [10,15]. Within the IV approach, one model in TSLS predicts treatment received *R* (the IO), given treatment assigned *T*, and a second model predicts outcome observed *Y*, given treatment received *R* (the IO). Both models are estimated jointly, to calculate accurate standard errors that account for the uncertainty in the first-stage model [15]. The TSLS allows the inclusion of covariates that predict treatment received *R*, and/or the outcome *Y*, which should increase the precision of the estimates [15]. Within this context it is possible to see that the term instrument represents the randomization to the treatment or control group (treatment assigned, *T*), which is assumed to influence treatment received, *R* (the IO) but not the outcome observed, *Y* (except through its effect on the IO treatment received); that is, the treatment assignment indicator, *T* (the instrument) is used in the model of the IO (*R*) but not in the model of the final outcome *Y*[15]. The specific models [15] are:

(5) $$\begin{array}{c} Y_{i}=\beta_{0}+\beta_{1}R_{i}+\theta X_{i}+e_{i}\\ R_{i}=\alpha_{0}+\alpha_{1}T_{i}+\phi W_{i}+v_{i} \end{array}$$

where *Yi* is the final outcome of interest, *Ti* is treatment assigned, *IOi* is treatment received (IO), the matrix *X* represents covariates that predict the outcome *Y*, and *W* consists of covariates that predict treatment received *R* (there may be overlap between the predictors in *X* and *W*). In the first model for *Yi* in [4] we do not use the observed *IOi* but the predicted *IOi* from the second model, which reduces the endogeneity of *IOi* and improves the prediction of *Yi*.

An intuitive rationale for the TSLS method in these contexts was given previously (formula (1)). Alternative estimation approaches include maximum likelihood [27] and Bayesian methods [28]. The TSLS technique is available in all main statistical packages.

### Truncation by death intermediate outcomes

The general approach considered under Section “Compliance as intermediate outcome” applies to truncation by death IOs. Now *IOi* denotes if subject *i* does or does not have the intermediate outcome, such that *IOi* (*Ti* =1) reflects the status of the IO in subject *i* if assigned to the experimental group and *IOi* (*Ti* =0) reflects the status of the IO in subject *i* if assigned to control. In this section we will assume that lack of compliance is not a problem in the trial.

The same labels for the four principal strata can be used, as (without loss of generalization), for example, compliers are considered as those who have the good IO when assigned to treatment and do not have it when assigned to control [25]. However, some of the key assumptions made in the case of noncompliance are usually not plausible in the case of truncation by death IOs. For example the monotonicity or no defiers assumption (assumption 4) could be controversial, as we could not rule out the existence of subjects not having the (good) IO when treated, while having it when not treated [25]. Furthermore, the exclusion assumption (assumption 5) is in general not sustainable, as it states that assignment to the (active) treatment does not have any effect on the stratum, always with the IO present (the AT stratum as previously described), and the stratum, always with the IO absent (the NT stratum). But if the final outcome *Y* is defined only when the IO is present, then the effect of assignment on *Y* in the AT stratum is precisely what we are interested in, so we cannot assume that the effect on this stratum is zero [25].

As previously noted, when all the five previously stated assumptions hold, the IV estimate is the simple treatment minus control estimate for the mean of *Y* divided by the simple treatment minus control estimate for the mean of intermediate outcome *R* (from formula (2)). So, even if we were able to accept all these assumptions when dealing with a truncation by death IO, we would require some imputation of the missing *Y* values for those subjects where *Y* is in fact not defined. As expected this arbitrary imputation usually results in arbitrarily distorted results [25]. We can then observe that in the case of truncation by death IOs, the simple estimation described in Section “Simple description of estimation” is in general not applicable, and additional or different assumptions should be made to be able to make a simple estimation. Alternatively more complex estimation approaches using covariates (as described in Section “More complex estimation”) must be used. Unfortunately, software for implementing PS are not readily available. A reference to one such program in R, PSpack, was made in 2004 [29] but we have not been able to locate it.

## Results

### Application to the calcium trial

Section “Noncompliance as intermediate outcome” will present the application of the methods described in Sections “Compliance as intermediate outcome”, “Assumptions underlying CACE analyses” and “Simple description of estimation” to the particular scenario of our calcium trial, where noncompliance is considered as an IO between calcium assignment and pregnancy as the (final) outcome. Section “Pregnancy as a “truncation by death” intermediate outcome” will illustrate the need for approaches beyond the standard PS approach (for example, IV) in the case of pregnancy as a truncation by death IO between calcium assignment and preeclampsia. Finally Section “Noncompliance and pregnancy as intermediate outcomes” will discuss how to account for both noncompliance and pregnancy as IOs in our calcium trial.

### Noncompliance as an intermediate outcome

From Tables 1 and 2 we see that if a subject is assigned to calcium and takes calcium, she can be a complier (C) or an always-taker (AT). If she is assigned to calcium and does not take calcium then she has to be a never taker (NT), as it is not possible to have defiers in our trial (D = Ø); the difference between never-takers and defiers might be slim, but it is essentially determined by the fact that our study is double-blinded, so subjects are told to do the same thing in both arms, that is, to take the pills assigned.

**Table 1:** Principal strata and sources of (non)compliance

	**Experimental group (calcium)**			**Control group (placebo)**		
**Source of (non) compliance**^**a**^	**Taking >80% of tablets assigned**	**Physician indicated collateral treatment involving substantial calcium**	**Subject self-indication collateral treatment involving substantial calcium**	**Taking >80% of tablets assigned**	**Physician indicated collateral treatment involving substantial calcium**	**Subject self-indication collateral treatment involving substantial calcium**
Strata						
Compliers	Yes	No	No	Yes	No	No
	Yes	Yes	No	Yes	Yes	No
	Yes	Yes	Yes	Yes	Yes	Yes
Always- takers	Yes	No	Yes	Yes	No	Yes
	No	Yes	No	No	Yes	No
	No	Yes	Yes	No	Yes	Yes
	No	No	Yes	No	No	Yes
Never-takers	No	No	No	No	No	No
Defiers*						

*As the trial is blinded, there are no defiers, as subjects cannot systematically take the opposite treatment. If a subject decides not to take the pills (treatment) assigned, she belongs to the never-takers stratum. Compliers are subjects who always take the treatment assigned.

^a^Sources of (non)compliance: 1, taking >80% of pills assigned; 2, collateral treatment involving substantial calcium indicated by physician; 3, self-indicated collateral treatment involving substantial calcium.

**Table 2:** **Principal strata according to potential behavior in each source of noncompliance (alternative version of Table**1**)**

**Strata**	**Behavior pattern in noncompliance sources 1, 2 and 3**^**a**^	**Treatment assigned and received for subject R in corresponding stratum**
Compliers	Yes, no, no	Ri(Ti) = Ti
	Yes, yes, no	
	Yes, yes, yes	
	Yes, no, yes	
Always takers	No, yes, no	Ri(Ti) = 1
	No, yes, yes	
	No, no, yes	
Never-takers	No, no, no	Ri(Ti) = 0
Defiers*		

*As the trial is blinded, there are no defiers, as subjects cannot systematically take the opposite treatment; **Ri = treatment received, Ti = treatment assigned (0 = placebo, 1 = calcium); ^a^sources of (non)compliance: 1, taking >80% of pills assigned; 2, collateral treatment involving substantial calcium indicated by physician; 3, self-indicated collateral treatment involving substantial calcium.

If a subject is assigned to placebo and takes placebo, she can be a complier (C) or a never-taker (NT); if she is assigned to placebo and takes calcium she has to be an always-taker (*AT*) as there are no defiers (D = Ø). Then from what we observe in the calcium arm we have estimations of *p*_*C+AT*_ and *p*_*NT*_, and from the placebo arm estimations of *p*_*C+NT*_ and *p*_*AT*_ ; combining these results we get two estimations for *p*_*C*_,

(6) $$\begin{array}{c} {\hat{p}}_{C1}={\hat{p}}_{C+\mathrm{AT}}-{\hat{p}}_{\mathrm{AT}}\\ {\hat{p}}_{C2}={\hat{p}}_{C+\mathrm{NT}}-{\hat{p}}_{\mathrm{NT}} \end{array}$$

and their (weighted) average *p*_*C*_ can be used as an estimation of the proportion of compliers in the population.

Assumption 1 (SUTVA) is sustained in our trial because the risk of pregnancy in woman *i* is not affected by the treatment received by women *j*, for all *j* ≠ *i*; as the treatment is administered at the level of the individual woman and the trial is double-blinded, there is nothing a particular woman or her physician can do to modify her outcome, from what they observe in other women in the trial. This assumption is required to be able to define an effect at the individual-woman level, which is implicit in the definition of the CACE.

Assumption 2 is sustained as the calcium trial is an RCT. Assumption 3 is sustained as we expect in fact that most of the subjects in both arms will comply. Assumption 4 (monotonicity) is sustained because as already discussed, the double-blinded design of the study prevents women from consistently taking the opposite treatment to the one assigned, so D = Ø. And finally assumption 5 (the exclusion restrictions) is sustained because the risk of pregnancy in our trial depends on calcium intake, not on calcium assignment; that is, given *IOi*, the risk of pregnancy is independent of *Ti*, and therefore, there is no effect of assignment on pregnancy for those who always behave the same (AT and NT strata).

Therefore, our calcium CACE can be estimated from the ITT effect and the estimated proportion of women in the compliers stratum (*p*_*C*_) as:

(7) $$\mathrm{CACE}=\frac{\mathrm{ITT}}{p_{C}}$$

### Pregnancy as a truncation by death intermediate outcome

In the calcium trial if compliance is (almost) perfect (for example, if for >95% of subjects *i*, *IOi* = *Ti*), we can concentrate on the effect of assignment to calcium on preeclampsia accounting for pregnancy as the only IO; and pregnancy is an IO which generates truncation by death, as the final outcome *Y,* preeclampsia is defined only for pregnant women (that is, when the IO is present).

As mentioned before, we do not expect calcium to influence the risk of pregnancy, but if we unfortunately observe a significant effect, we will have to consider pregnancy as an IO and account for it properly. In this case we will label the relevant causal effect as pregnancy average causal effect, or PACE, as we are only interested in the possible effect of assignment to calcium on preeclampsia among women who get pregnant within the trial (the AT stratum), now better labeled as the always-pregnant stratum).

Some of the assumptions made in Section “Assumptions underlying CACE analyses” to estimate the CACE (as in Section “Noncompliance as intermediate outcome”) are now inappropriate, and some new assumptions are relevant and possibly convenient. For example we have just seen that assumption 5 (the exclusion-restriction assumptions) is generally unsustainable, because we are usually interested in the effect on those who have always the IO present (that is, the AT or always-pregnant stratum), which then cannot be assumed to be zero. On the other hand, additional assumptions now make sense. For example in our trial calcium could either increase or decrease the risk of pregnancy, but not both; if calcium does decrease the risk of pregnancy (which might be the case if there is any effect at all), then we might question the compliers stratum, that is, we could assume that there are no women who get pregnant under calcium or under placebo. We will subsequently consider this in more detail, where the two IOs, compliance and pregnancy, will be combined and a simple estimate will be pursued. One of the main reasons for the differential sustainability of the set of assumptions between compliance and pregnancy as IOs is that compliance is an IO quite connected to the behavior of individuals, and to some extent under their subjective control, while pregnancy is not. In other words, (lack of) compliance can be heavily addressed using social sciences tools, while (lack of) pregnancy cannot.

If revising and making additional assumptions do not make the simple estimate feasible we can alternatively use the TSLS technique as previously described to address the problem of pregnancy as an IO. The relevant issue in this case would be then to identify relevant covariates for the model of *R* (IO pregnancy) given *T* (treatment assigned), and for the model of outcome *Y* (preeclampsia) given *R*, that is, matrix *W* and *X* respectively (see expression (4) in Section “More complex estimation”). From the baseline form of our calcium trial we identified several potential good predictors of pregnancy and of preeclampsia, which are presented in Table 3 and will eventually be used in the TSLS analysis.

**Table 3:** Covariates for the two-stage least squares (TSLS) analysis

**Regression model for pregnancy, given treatment assignment:**	
**Questions on the form: Description of the covariate:**	
ADM_Q2	Age (years)
ADM_Q3	Parity (previous pregnancies >24 weeks)
ADM_Q14	Any health problems
ADM_Q1SA	Blood pressure systolic (normal/abnormal)
ADM_Q15B	Blood pressure diastolic (normal/abnormal)
ADM_Q16	Body mass index (normal/abnormal)
ADM_Q17	
**Regression model for preeclampsia. Given pregnancy:**	
Questions on the form: Description of the covariate:	
ADM_Q2	Age (years)
ADM_Q3	Parity (previous pregnancies >24 weeks)
ADM_Q14	Any health problems
ADM_Q1SA	Blood pressure systolic (normal/abnormal)
ADM_Q1SB	Blood pressure diastolic (normal/abnormal)
ADM_Q16	Body mass index (normal/abnormal)
ADM_Q17	

### Noncompliance and pregnancy as intermediate outcomes

When we combine compliance and pregnancy as IOs between calcium assignment and preeclampsia we get 16 principal strata as a result of combining the four principal strata of noncompliance with the corresponding four of pregnancy (see Table 4). The insertion of pregnancy as a truncation by death IO means that the (average treatment assignment) effects in many of the strata are not defined (12 out of 16), as the effect of calcium assignment on preeclampsia is defined only for strata where women get pregnant under both arms of the trial (always-pregnant strata 7, 12, 14 and 16). In fact within these four strata where the effect is defined, in two (strata 7 and 16) the effect is zero because of the exclusion restrictions assumption (assumption 5, Section “Assumptions underlying CACE analyses”); and finally the effect for stratum 14 does not influence the global ITT effect, as this stratum is empty (no defiers or monotonicity assumption 4, Section “Assumptions underlying CACE analyses”). Therefore the global ITT effect, computed effectively over strata 7, 12, 14 and 16, reduces to:

(8) $$\begin{array}{l} \tau =\frac{1}{N}\overset{N}{\underset{i=1}{\sum }}Y_{i}\left(T_{i}=1,P_{i}\right)-Y_{i}\left(T_{i}=0,P_{i}\right).\\ \;=p_{1}\tau_{1}+p_{2}\tau_{2}+\ldots +p_{16}\tau_{16}\\ \;=p_{7}\tau_{7}+p_{12}\tau_{12}+p_{14}\tau_{14}+p_{16}\tau_{16}\\ \;=p_{12}\tau_{12}. \end{array}$$

**Table 4:** Principal strata for both compliance and pregnancy as IOs

	**Treatment assigned**					**Sample space of final outcome: preeclampsia**			
	**Control**		**Treatment**						
**Principal strata**	**Treatment**	**Pregnant (0 = placebo; 1 = calcium)**	**Treament received (0 = no; 1 = yes)**	**Pregnant (0 = no; 1 = yes)**	**Labels**	**Assigned to control**	**Assigned treatment**	**Proportion in the stratum**	**(Average treatment assigned ) Effect**
1	0	0	0	0	Never-takers never pregnant	*	*	p1	*
2	0	0	0	1	Never-takers pregnant under treatment	*	(no, yes)	p2	*
3	0	0	1	0	Compliers never-pregnant	*	*	p3	*
4	0	1	0	0	Never-takers pregnant under control	(no, yes)	*	p4	*
5	1	0	0	0	Defiers never-pregnant	*	*	0	*
6	0	0	1	1	Compliers pregnant under treatment	*	(no, yes)	p6	*
7	0	1	0	1	Never-takers always-pregnant	(no, yes)	(no, yes)	p7	0
8	1	0	0	1	Defiers pregnant under treatment	*	(no, yes)	0	*
9	0	1	1	0	Compliers pregnant under control	(no, yes)	*	p9	*
10	1	0	1	0	Always-takers never-pregnant	*	*	p10	*
11	1	1	0	0	Defiers pregnant under control	(no, yes)	*	0	*
12	0	1	1	1	**Compliers always-pregnant**	(no, yes)	(no, yes)	p12	τ12
13	1	0	1	1	Always-takers pregnant under treatment	*	(no, yes)	p13	*
14	1	1	0	1	Defiers always-pregnant	(no, yes)	(no, yes)	0	τ14
15	1	1	1	0	Always-takers pregnant under control	(no, yes)	*	p15	*
16	1	1	1	1	Always-takers always-pregnant	(no, yes)	(no, yes)	p16	0

*Final outcome or effect not defined; **it is possible in theory to have always-takers, because women can take calcium supplementation out of the trial intervention.

Then to solve for *τ*_12_ in [8] we only need to have an estimate of *p*_12_.

In Table 5 we present the groups that can be observed in our calcium trial and the corresponding principal strata behind them (from Table 4), which clearly illustrates the identification problem. Under the current assumptions we see that the stratum we want to identify (stratum 12) is linked to other three (nuisance) strata both in the OBS(111) group (women randomized to calcium, receiving calcium, and pregnant) and in the OBS (011) group (women randomized to placebo, receiving calcium, and pregnant).

**Table 5:** Observed groups (according to treatment assigned received and pregnancy status) and corresponding principal strata

	**Description**	Principal strata under initial assumptions^*^	Principal strata under additional assumption^**^
Randomized to calcium:			
OBS(100)	received placebo and non pregnant	1, 4	NA
OBS(101)	received placebo and pregnant	2, 7	NA
OBS(110)	received placebo and non pregnant	3, 9, 10, 15	3, 9, 10, 15
OBS(111)	received placebo and pregnant	6, 12, 13, 16	6, 12, 13, 16
Randomized to Placebo:			
OBS(000)	received placebo and non pregnant	1, 2, 3, 6,	3, 6
OBS(001)	received placebo and pregnant	4, 7, 9, 12	9, 12
OBS(010)	received placebo and non pregnant	10, 13	10, 13
OBS(011)	received placebo and pregnant	15, 16	15, 16

OBS (ijk), observed women randomized to i (0 = placebo, 1 = calcium), receiving j (0 = placebo, 1 = calcium), and pregnant k (0 = no, 1 = yes). ^*^Initial assumptions are assumptions 1 to 5 in Section “Noncompliance as intermediate outcome”; ^**^additional assumptions a) and b) described in Section “Noncompliance and pregnancy as intermediate outcomes”.

NA, the corresponding principal strata are empty under additional assumptions.

It is then obvious that we need to make reasonable additional assumptions if we want to obtain a simple estimate of the causal effect of calcium on preeclampsia. First, it might make sense to additionally assume that

a) the never-takers (NT) stratum is empty, as women recruited into the trial are not mentally sick, and will be properly informed about the potential advantages and disadvantages of calcium supplementation; in this scenario to have never-takers could be considered close to impossible. This new reasonable assumption quite simplifies the identification process, as now strata 1, 2, 4 and 7 are all empty (that is, *p*_1_ = *p*_2_ = *p*_4_ = *p*_7_ = 0 ).We can also make more precise our assumptions in relation to the possible effect of calcium on pregnancy. Calcium intake can affect pregnancy either by increasing or by decreasing its risk, and we could (additionally) assume that if any,

b) calcium increases the risk (probability) of pregnancy. This means that the compliers stratum with women getting pregnant with placebo and not with calcium (stratum 9 in Table 4) is empty (that is, *p*_9_ = 0). These additional assumptions a) and b) allow us to identify the number of subjects in stratum 12 from the number of subjects observed in OBS(001), so we can estimate *p*_12_ and then *τ*_12_ from (7). Complementary assumptions, a1) and a2):

a) a1) the always-takers (AT) stratum is empty, and

b) b1) calcium decreases the risk (probability) of pregnancy,

could be made, which would also allows us to identify (the number of subjects in) stratum 12 from the number of subjects in OBS(111), and then estimating *p*_12_ and finally *τ*_12_ from (7). Assuming a1) in addition to a) is plausible, but obviously b1) and b) exclude each other.

## Discussion

In this paper we have presented an introduction to the PS approach and how it can be applied to account for IOs in RCTs. Some other papers have further discussed its assumptions and limitations, as well as alternative approaches [15,23]. Here we would instead like to focus on why we really need to move beyond the (modified) ITT approach when dealing with IOs, and to debate on some of the obstacles possibly blocking the way out.

First of all, there is an increasing trend to label trials as pragmatic under the assumption that they are more relevant to public health decision makers (PHDM); this usually comes together with the attractive statement that in pragmatic trials a comprehensive and consistent ITT approach is just what is needed. It would seem then that the resulting combined package is unbeatable. However, some pragmatic trials are really hybrid, and then imply an interest in knowing why the experimental intervention does (or does not) work, as this information could be required by PHDM when implementing trial results in different contexts (in fact the causal effect is considered by some as more generalizable than the ITT effect [15]. This means that on some occasions pragmatic trials include an explanatory component related to the causal effect of the intervention tested (considered complex but relevant [14,30]), or related to effects estimated on other subgroups of patients defined by alternative IOs (for example, women with complete follow up, women who got pregnant during the trial, et cetera). And this means that on occasions the ITT approach is not the answer for all the relevant questions.

The CONSORT 2010 guidelines [31] widely recommend the use of the ITT approach but do not make explicit mention of IOs; two particular cases of IOs, noncompliance and missing data, are mentioned but no details are given on how to address them, which is expected as these guidelines attempt to give advice on the reporting of what was done and not to judge on particular methods. In the case of noncompliance the CONSORT guidelines say: ‘Noncompliance with assigned therapy may mean that the intention-to-treat analysis underestimates the potential benefit of the treatment, and additional analyses, such as a per protocol analysis, may therefore be considered. It should be noted, however, that such analyses are often considerably flawed.’ In the case of missing data CONSORT says that ‘Participants with missing outcomes can be included in the analysis only if their outcomes are imputed’; in fact, consequent application of the ITT approach requires imputation of missing data.

Some researchers conclude from the above CONSORT recommendations that the ITT approach can properly account for IOs, as alternative approaches are rather obscure or dubious. In fact, one of the unintended problems of the ITT approach is that in some contexts it has allowed counterproductive and excessive relaxation to flourish. Labeling a trial as having an ITT approach is seen by some as a quality guarantee or safe haven, which has been proved not to be the case at all [14]. Some researchers also believe that the ITT approach is universally applicable, not noting that full application of the ITT approach is possible only when complete outcome data are available for all randomized subjects [14], this being relevant both for lost to follow up data and for truncation by death IOs. Some suggestions have been presented to minimize losses to follow up and the corresponding negative impact of missing data [30] but they do not apply to truncation by death IOs, and to some extent are not in the spirit of pragmatic trials.

Unfortunately it has become common practice to consider that pragmatic trials (and the corresponding ITT approach), can emphasize on wide generalizability at the expense of methodological rigor, but this ‘…can result in invalid and unreliable results. Achieving a creative tension between the two is crucial’ [32]. Particularly in relation to treatment compliance, it ‘…is one of the most important outcomes of pragmatic trials. Unlike explanatory trials where compliance with the intervention must be ensured in order to know that the intervention can work, in pragmatic trials compliance with the intervention is measured as an outcome’; and: ‘Compliance is not something you necessarily struggle to maintain but rather something you measure as an outcome. Lack of compliance in the “real world” frequently renders an efficacious intervention ineffective’ [33].

## Conclusions

We hope that this paper and discussion will help researchers to be critical about the limitations of the ITT approach to account properly for the quite common presence of IOs in RCTs, and that possibly more complex but definitely more appropriate approaches be better known and eventually applied. The sound basis of the ITT approach is widely appreciated, but we should also recognize that it does not fit all problems and that on occasions, as illustrated in this paper it should be complemented by alternative approaches.

## Abbreviations

AT: Always-taker; C: Complier; CACE: Compliers average causal effect; D: Defier; ITT: Intention-to-treat; IO: Intermediate outcomes; IV: Instrumental variables; NT: Never-taker; PACE: Pregnancy average causal effect; PHDM: Public health decision makers; PS: Principal stratification; RCT: Randomized clinical trial; SUTVA: Stable unit treatment value assumption; TSLS: Two-stage least squares; WHO: World Health Organization.

## Competing interests

The authors declare that they have no competing interests.

## Authors’ contributions

AHS developed the original concept for the study, with input from AP. All authors contributed to the paper during development and read and approved the final version of the manuscript.

## Authors’ information

AHS and AP are statisticians, and APB and AMG are Medical Officers at the Reproductive Health Research Department, WHO, Geneva.
